# Identification of a Large* SLC25A13* Deletion via Sophisticated Molecular Analyses Using Peripheral Blood Lymphocytes in an Infant with Neonatal Intrahepatic Cholestasis Caused by Citrin Deficiency (NICCD): A Clinical and Molecular Study

**DOI:** 10.1155/2016/4124263

**Published:** 2016-04-05

**Authors:** Qi-Qi Zheng, Zhan-Hui Zhang, Han-Shi Zeng, Wei-Xia Lin, Heng-Wen Yang, Zhi-Nan Yin, Yuan-Zong Song

**Affiliations:** ^1^Department of Pediatrics, The First Affiliated Hospital, Jinan University, Guangzhou 510630, China; ^2^Core Laboratory, The First Affiliated Hospital, Jinan University, Guangzhou 510630, China; ^3^Biomedical Translational Research Institute, Jinan University, Guangzhou 510630, China

## Abstract

*Background.* Neonatal intrahepatic cholestasis caused by citrin deficiency (NICCD) is a Mendelian disorder arising from biallelic* SLC25A13 *mutations, and* SLC25A13* genetic analysis was indispensable for its definite diagnosis. However, conventional* SLC25A13 *analysis could not detect all mutations, especially obscure large insertions/deletions. This paper aimed to explore the obscure* SLC25A13* mutation in an NICCD infant.* Methods. *Genomic DNA was extracted to screen for 4 high-frequency* SLC25A13* mutations, and then all 18 exons and their flanking sequences were analyzed by Sanger sequencing. Subsequently, cDNA cloning, SNP analyses, and semiquantitative PCR were performed to identify the obscure mutation.* Results. *A maternally inherited mutation IVS16ins3kb was screened out, and then cDNA cloning unveiled paternally inherited alternative splicing variants (ASVs) featuring exon 5 skipping. Ultimately, a large deletion c.329-1687_c.468+3865del5692bp, which has never been described in any other references, was identified via intensive study on the genomic DNA around exon 5 of* SLC25A13* gene.* Conclusions. *An NICCD patient was definitely diagnosed as a compound heterozygote of IVS16ins3kb and c.329-1687_c.468+3865del5692bp. The large deletion enriched the* SLC25A13* mutation spectrum, and its identification supported the concept that cDNA cloning analysis, along with other molecular tools such as semiquantitative PCR, could provide valuable clues, facilitating the identification of obscure* SLC25A13* deletions.

## 1. Introduction

Human citrin deficiency is an autosomal recessive disease due to dysfunction of citrin, the liver-type calcium-stimulated aspartate-glutamate carrier isoform 2 (AGC2) encoded by the* SLC25A13* gene [1, 2]. Up to now, three age-dependent clinical phenotypes had been reported for this disorder, that is, adult-onset citrullinemia type 2 (CTLN2) in adolescents and adults, neonatal intrahepatic cholestasis caused by citrin deficiency (NICCD) in neonates or infants, and failure to thrive and dyslipidemia caused by citrin deficiency (FTTDCD) in older children [3–8]. Although most reported patients were Asian [9–14], citrin deficiency has been recognized as a worldwide panethnic disorder nowadays [15–19].

Due to the lack of well-recognized clinical/biochemical diagnostic criteria for NICCD,* SLC25A13 *genetic analysis has been regarded as reliable tool for the definite diagnosis of such patients. However, routine* SLC25A13* genetic analyses, such as polymerase chain reaction (PCR), long and accurate-PCR (LA-PCR), PCR-restriction fragment length polymorphism (RFLP), and Sanger sequencing, could not detect all* SLC25A13 *mutations, especially large deletions or insertions of an obscure nature. It has been estimated that approximately 15% of compound heterozygotes or homozygotes carrying* SLC25A13* mutations in both alleles could not be definitely diagnosed just by the above conventional approaches [20]. In such cases, other molecular tools, although usually labor-intensive and cost-expensive, were needed to identify the obscure mutations.

In this study, a large* SLC25A13* deletion was identified via sophisticated molecular analyses using peripheral blood lymphocytes (PBLs) in an NICCD patient, who responded well to a lactose-free and medium-chain triglycerides- (MCTs-) enriched formula. We herein reported the clinical and molecular findings.

## 2. Subjects and Methods

### 2.1. Subjects and Ethics

The research subjects in this study were a male patient suspected to have NICCD and his parents as well. With the informed consent from the parents and the ethical approval by the medical ethical committee of our hospital, we performed intensive clinical and genetic study on this family.

### 2.2. Conventional DNA Analysis

The DNA was extracted from the peripheral venous blood following the genomic DNA extraction kit (Omega, USA) instructions. Four high-frequency* SLC25A13* mutations, c.851_854del, c.1638_1660dup, IVS6+5G>A, and IVS16ins3kb, were screened by PCR, LA-PCR, and PCR-RFLP procedures, and then Sanger sequencing of all the 18 exons and their flanking sequences was undertaken, using direct sequencing of DNA fragments amplified by genomic DNA-PCR to identify novel mutation in the gene* SLC25A13 *[21].

### 2.3. Reverse Transcriptional PCR (RT-PCR) and Nested PCR

PBLs were separated using lymphocyte separation medium (LSM, MP) from 2-3 milliliters of ethylene diamine tetraacetic acid- (EDTA-) anticoagulant peripheral venous blood [20]. PBLs were cracked with ribonucleic acid (RNA) trizol reagent (The Life Technologies) and total RNA was extracted using a variety of organic solvents, as described previously [12, 22–25]. The cDNAs were synthesized from total RNA by Moloney murine leukemia virus (MMLV) reverse transcriptase (TaKaRa). Nested PCR was used to amplify* SLC25A13* open reading frame (ORF), and the primers and PCR temperature profile were described in detail as in previous references [21, 22]. The two primer pairs in Nested PCR were RAS2 and RACEA1 in the first PCR and RAS3 and Ex18R in the second one, with expected PCR products of 3107 bp and 2191 bp in size, respectively. Both of them contained the citrin-coding sequence (CDS).

### 2.4. Molecular Cloning and Alternative Splicing Variants (ASVs) Analyses

The Nested PCR products of paternal origin were purified by using a gel extraction kit (Omega) and then connected with the sequence of the pMD 18-T vector (TaKaRa) and transformed into DH5*α Escherichia coli* competent cells, as in our previous publications [12, 22, 24, 25]. Following that, the transformed cells were cultured in the shaking table for 4 h and then coated on plates after centrifugation on 1000 rpm for 5 min. After cultivation in 37°C for 16 hours, the positive clones which had the targeted bands after PCR amplification were selected and sequenced. The ASV sequences were analyzed by using the ContigExpress and DNAMAN software, and their designation was according to the nomenclature guidelines [26, 27].

### 2.5. Further Location and Identification of the Obscure Mutation

**Figure 1:**
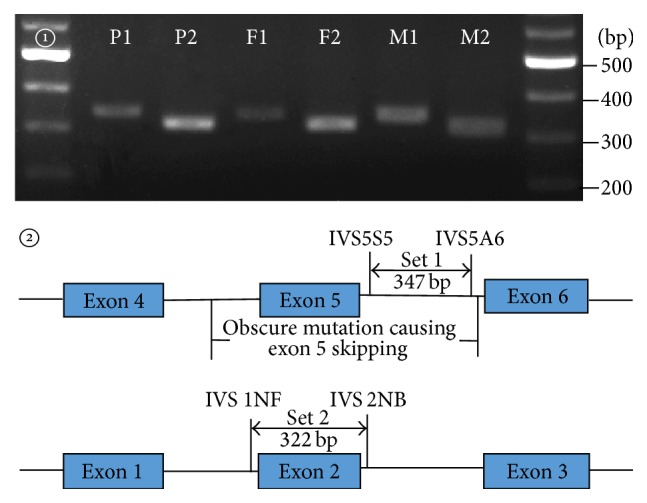
Semiquantitative PCR in positioning analysis of the novel large deletion. Figure 1① was a representative electrophoresis of the semiquantitative PCR products. Compared with the mother (M1), the patient (P1) and the father (F1) had less signal intensity of the PCR products when using primer Set 1, while this is not the case when using primer Set 2; note that the signal intensity of the PCR products in the patient (P2) and the father (F2) was similar to that in the mother (M2). Figure 1② depicted the positions of the primer Sets 1 and 2. The primer sequences in Set 1 were 5′-GAGCTTCTTAGAAACCACCATGTGG-3′ (IVS5S5) and 5′-TCCAATGAGG AAGAAGACTACAGGAAG-3′ (IVS5A6), while in Set 2, 5′-TTTATGCACTGGGGCAACATG-3′ (IVS 1NF) and 5′-TGCCGGGCTGACACTTTGG-3′ (IVS 2NB), respectively. The results suggested that the patient (P) and the father (F) might harbor an obscure large deletion around the primer Set 1 but not Set 2.

According to the ASV structures, to further locate the DNA span around exon 5 that might contain the obscure mutation, semiquantitative PCR was performed in a total volume of 50 *μ*L, containing 5 *μ*L of 10x Buffer (Mg^2+^ plus) (TaKaRa), 4 *μ*L of dNTP (10 mM), 37.75 *μ*L of sterilized distilled water, 0.25 *μ*L of Taq (TaKaRa), 2 *μ*L of the forward and reverse primers together, and 1 *μ*L of DNA template. Then, all tubes were placed into a thermal cycler and the parameters were set in 94°C for 5 minutes followed by 28 to 30 cycles of 94°C for 30 seconds, 60°C for 40 seconds, 72°C for 40 seconds, and a final extension step at 72°C for 10 minutes. The two primer pairs were IVS5S5 and IVS5A6 in Set 1 and IVS 1NF and IVS 2NB in Set 2, whose sequences and locations were displayed in Figure 1, respectively.

Based on the findings above, a PCR approach using the primers IVS4S3 and J5.6KbDelR1 (Set 3) was carried out to identify the obscure mutation. The reaction mixture was a total volume of 50 *μ*L, containing 5 *μ*L of 10x LA Buffer (Mg^2+^ plus) (TaKaRa), 8 *μ*L of dNTP (10 mM), 30.3 *μ*L of sterilized distilled water, 0.2 *μ*L of Taq, 0.5 *μ*L of LA Taq (TaKaRa), 4 *μ*L of the forward and reverse primers together, and 2 *μ*L of DNA template. The temperature profile was 95°C for 5 minutes followed by 40 cycles of 94°C for 40 seconds, 55°C for 40 seconds, 68°C for 10 minutes, and a final extension step at 68°C for 10 minutes.

### 2.6. Statistical Analysis

The proportions of the ASVs with exon 5 skipping in all ASVs from the patient and 8 healthy volunteers were statistically assessed using SPSS (Version 13.0) chi-square test, and a *P* value of less than 0.05 was considered statistically significant.

## 3. Results

### 3.1. Clinical Findings

A male infant at the age of 2 months was referred to the local hospital with the chief complaint of jaundiced skin over 10 days. Physical examination at referral revealed a mildly pale face and hemorrhagic spots scattered on the skin of whole body. The lungs were clear on auscultation. No abnormal sound or murmur was heard on heart auscultation. An enlarged liver, 5.0 cm below the right costal margin, was palpated. No pathological reflexes could be found on nervous system examination. Slightly visible pitting edema could be found in both lower extremities.

Biochemical test demonstrated elevated aspartate aminotransferase (AST), gamma-glutamyl transpeptidase (GGT), total bilirubin (Tbil), direct bilirubin (Dbil), indirect bilirubin (Ibil), and total bile acids (TBA), which indicated intrahepatic cholestasis. The albumin level was decreased while that of alpha-feto protein (AFP) elevated markedly (Table 1). Ultrasonography revealed hepatomegaly, ascites, and abdominal bloating, along with a patent oval foramen of the heart. The urinary gas chromatography mass spectrometry (GC-MS) analysis discovered large quantity of hexanedioic acid and 4-hydroxyphenyl lactate (4HPL), and on tandem mass spectrometry (MS-MS) analysis, elevated levels of citrulline and arginine were detected.

NICCD was thus suspected based on the above clinical and biochemical findings, and according to the pediatrician's advice, breast feeding was stopped and a lactose-free and MCT-enriched therapeutic formula was introduced immediately. In the subsequent follow-up, the clinical manifestations were alleviated gradually. At his age of 3.7 months, the infant was referred to our hospital to confirm the NICCD diagnosis by* SLC25A13 *gene analyses.

### 3.2. Screening and Sequencing Results

Screening of the 4 high-frequency mutations proved that the patient harbored a maternally inherited mutation IVS16ins3kb, but another mutation was not detected. Even after Sanger sequencing of all the 18 exons and their flanking sequences, the* SLC25A13* mutation of paternal origin remained obscure. Therefore, cDNA cloning analysis was undertaken to facilitate the identification of the obscure mutation.

### 3.3. Findings of cDNA Cloning Analysis

As the maternal mutation IVS16ins3kb led to production of an aberrant mRNA molecule with a new exon 17, losing the normal exons 17 and 18 [21], only the ASVs transcribed from the paternal* SLC25A13* allele were amplified by the Nested PCR approach using the two reverse primers both within the normal exon 18 of* SLC25A13 *gene. In other words, the 26 ASVs in Table 2 all resulted from the paternally inherited* SLC25A13* allele.

Similar to our previous findings [22], these ASVs demonstrated remarkable structural heterogeneity. However, all these ASVs featured exon 5 skipping (*r*.329_468del), as shown in Table 2. When compared with the ASVs in the 8 healthy volunteers which had been reported previously [24], exon 5 skipping was a unique structural feature of the ASVs in this patient (*χ* ^2^ = 129.2 and *P* < 0.01 as in Table 3), strongly suggesting the existence of a large insertion or deletion around exon 5 at the DNA level.

### 3.4. Positioning and Sequencing Analysis of the Obscure Mutation

**Figure 2:**
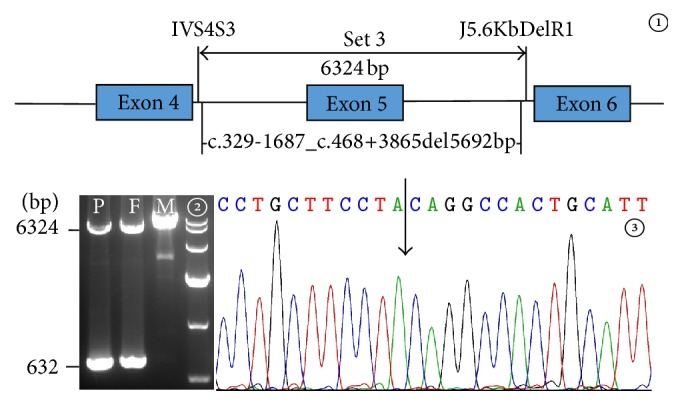
The large deletion mutation in* SLC25A13* gene of the infant and his father. Figure 2① depicted the positions of the primer Set 3 and the novel large deletion. The primer sequences were 5′-AAGATTGTTGTTTATGGTGAGAC-3′ for IVS4S3 and 5′-ATGGTTTGCCCGACATGAGTAATC-3′ for J5.6KbDelR1, respectively. In Figure 2②, LA-PCR with the primer Set 3 revealed that the patient (P) and his father (F), but not the mother (M), had an unexpected band of 632 bp in size besides the normal product of 6324 bp. Figure 2③ was a segmental sequencing result of the unexpected PCR product. The arrow indicated the breakpoint arising from the large deletion.

Based on the findings above, a diversity of primers was designed and PCR amplification conducted to analyze a variety of SNPs within the DNA fragment around exon 5 in all 3 family members. Although no clues suggestive of the obscure mutation could be detected by these analyses, semiquantitative PCR had positive findings—when amplified using the primer Set 1 near exon 5, the DNA samples of the patient (P1) and the father (F1) had less PCR products in comparison to that of the mother (M1), but this is not the case when using the primer Set 2 covering the entire exon 2 (Figure 1), indicating that the patient and the father might have a large deletion or insertion involving the positions of the primer Set 1. According to this result, LA-PCR amplification using the primer Set 3 (Figure 2) yielded an unexpected band of 632 bp in size in the patient and father, besides the expected 6324 bp product as in the mother, and subsequent Sanger sequencing of the unexpected product uncovered a large deletion c.329-1687_c.468+3865del5692bp.

## 4. Discussion

Although a maternally originated* SLC25A13* mutation IVS16ins3kb had been discovered as a high-frequency mutation in this study, conventional genetic analyses, such as PCR/LA-PCR, PCR-RFLP, and Sanger sequencing, could not unveil the paternally inherited mutation in the infant with typical clinical and biochemical features of intrahepatic cholestasis, making the definite diagnosis of NICCD a challenge. It was by using cDNA cloning along with SNP analysis and semiquantitative PCR in all family members that c.329-1687_c.468+3865del5692bp, a large deletion, was finally identified, which has never been described in any other references. Although time- and cost-consuming, the identification of this novel deletion confirmed the feasibility of* SLC25A13* cDNA cloning analysis using PBLs as a molecular tool facilitating the identification of large deletions and provided reliable evidence for the definite diagnosis of NICCD in the patient along with the mutation IVS16ins3kb of maternal origin. This was important not only for the proband himself but also for future genetic counseling and antenatal diagnosis in the family.

This 5692 bp deletion resulted in exon 5 skipping at mRNA level, as described in Table 2, theoretically yielding a truncated citrin protein of 127 amino acids—the exon 5 skipping gave rise to a frameshift at codon 110, continued to translate 17 amino acids, and then encountered a terminator codon at the position of 127, eventually producing an aberrant citrin molecule p.E110fs127X. Interestingly, this truncated citrin molecule was the same as the one arising from the large insertion IVS4ins6kb which had been reported very recently by our group [24]. The aberrant mRNA with exon 5 skipping could be a target for nonsense-mediated mRNA decay (NMD). Moreover, according to the molecular structural feature of mitochondrial AGCs (aspartate/glutamate carriers) [28], such a truncated citrin molecule predictively lost the entire mitochondrial carrier domain and had no AGC2 function to export aspartate and import glutamate together with a proton into the mitochondrial matrix, thus causing a series of metabolic disturbances and finally leading to the formation of the laboratory and clinical presentations in the patient.

The lactose-free and MCT-enriched formulas have been reported to be clinically and biochemically effective for NICCD patients in increasing clinical cases [12, 25, 29–31]. The clinical process in this study further supported this concept. Overall, the increased cytosolic NADH/NAD^+^ ratio in hepatocytes has been well-recognized as a key biochemical alteration for citrin deficiency [6]. Breast milk or common formula contained high carbohydrate in the form of lactose, a well-known disaccharide that was easily digested by lactase on the intestinal mucosa into glucose and galactose, both of which were then rapidly absorbed into the blood flow. In hepatocytes, galactose was conversed via Leloir pathway into glucose [32], the metabolism of which produced NADH, and thus increased the cytosolic NADH/NAD^+^ ratio in the liver and caused liver damage in patients with citrin deficiency [6, 31]. Moreover, secondary galactosemia due to citrin deficiency might be involved in the NICCD pathogenesis—the increased cytosolic NADH/NAD^+^ ratio in citrin-deficient hepatocytes might inhibit the activity of uridine diphosphate- (UDP-) galactose-4-epimerase [33], leading to accumulation of a large quantity of galactitol and galactonate [34], and galactitol has been suggested as one of the substrates causing jaundice, hepatosplenomegaly, hepatocellular insufficiency, and cataracts [30, 35, 36].

Energy shortage in the liver caused by an impairment of glycolysis due to an increased NADH/NAD^+^ ratio has been proposed as an important pathophysiology in citrin deficiency [31, 37]. Since the galactose kinase reaction in Leloir pathway was energy-consuming [32], the galactose metabolism in hepatocytes inevitably exacerbated such a pathophysiology. On the other hand, the absorption of MCTs was not bile acid-dependent, which might reduce the burden of the liver to synthesize and excrete bile salt into the gut [30]. Of particular note, MCTs can be better absorbed and transported via the portal vein into the liver and were mainly metabolized to acetyl-CoA along with FADH_2_ and NADH, which could supply more such substrates to hepatic cells as energy sources [31]. Taking all these factors together, it was not surprising for the NICCD infant in this study to respond well to the lactose-free and MCT-enriched therapeutic formula.

In summary, by sophisticated molecular analysis using PBLs, this study definitely diagnosed an NICCD patient who was a compound heterozygote of the IVS16ins3kb mutation and a novel deletion c.329-1687_c.468+3865del5692bp in the* SLC25A13* gene. The large deletion constituted a novel component in the* SLC25A13* mutation spectrum, and its identification lent further support to the concept that* SLC25A13* cDNA cloning analysis using PBLs, along with other molecular tools such as semiquantitative PCR, could provide valuable clues, facilitating the identification of unknown large deletions.

## Figures and Tables

**Table 1:** Biochemical changes over time in the NICCD infant.

Biochemical indices	2.0M	2.3M	2.4M	2.5M	2.6M	2.8M	3.0M	3.7M^a^	4.8M	8.8M	17.7M
ALT (5–40 U/L)	46.00	29.80	40.39	34.48	33.00	30.00	19.23	20.00	174.00	63.00	27.00
AST (5–40 U/L)	83.00	127.50	116.92	73.47	72.00	62.00	45.02	37.00	172.00	54.00	30.00
GGT (8–50 U/L)	—	—	131.13	139.63	183.00	185.00	475.33	211.00	109.00	54.00	26.00
ALP (20–500 U/L)	—	—	246.65	205.63	259.00	201.00	269.91	385.00	418.00	243.00	251.00
TP (60.0–83.0 g/L)	33.90	66.20	67.53	66.32	62.80	63.70	65.39	54.40	61.70	66.00	62.80
Alb (35.0–55.0 g/L)	21.30	36.94	39.66	39.23	35.00	37.60	40.87	37.50	41.80	45.80	43.80
Glb (20.0–30.0 g/L)	—	29.26	27.87	27.09	27.80	26.10	24.52	16.90	19.90	20.20	19.00
Tbil (2–19 *μ*mol/L)	178.60	163.09	172.99	92.56	56.50	60.90	63.61	38.40	44.70	3.20	6.20
Dbil (0–6 *μ*mol/L)	70.70	66.76	79.86	48.95	35.10	31.90	35.04	31.00	36.30	1.00	2.00
Ibil (2.56–20.9 *μ*mol/L)	107.9	96.33	93.13	43.61	21.40	29.00	28.57	7.40	8.40	2.20	4.20
TBA (0–10 *μ*mol/L)	—	33.86	39.35	54.43	—	73.50	44.45	173.60	169.7	11.30	8.80
AFP(0–12 ng/mL)	—	—	—	—	—	—	—	10709.20	887.20	—	1.90

ALT, alanine aminotransferase; AST, aspartate aminotransferase; GGT, *γ*-glutamyl transpeptidase; ALP, alkaline phosphatase; TP, total protein; Alb, albumin; Glb, globulin; Tbil, total bilirubin; Dbil, direct bilirubin; Ibil, indirect bilirubin; TBA, total bile acid; AFP, alpha-feto protein. M, months of age. —, not tested. ^a^When the breast milk feeding was stopped while a galactose-free and MCT-enriched therapeutic formula was introduced.

**Table 2:** The *SLC25A13* ASVs detected by cDNA analysis in the NICCD patient.

Clones	Alternative splicing variants (ASVs)	Annotations
C0282-1	*r*.213_468del; *r*.1194A>G	Exons 4 and 5 skipping
C0282-2	*r*.70_468del; *r*.1194A>G	Exons 3, 4, and 5 skipping
C0282-3	*r*.70_468del; *r*.1194A>G	Exons 3, 4, and 5 skipping
C0282-4	*r*.213_468del; 1311_1312ins1311+102_1312+176; *r*.1194A>G	Exons 4 and 5 skipping
C0282-5	*r*.213_468del; *r*.616_848del; *r*.1194A>G	Exons 4, 5, 7, and 8 skipping
C0282-6	*r*.70_468del; *r*.1194A>G	Exons 3, 4, and 5 skipping
C0282-7	*r*.329_468del; *r*.1194A>G	Exon 5 skipping
C0282-8	*r*.213_468del; *r*.755_848del; *r*.1194A>G	Exons 4, 5, and 8 skipping
C0282-9	*r*.329_468del; *r*.1194A>G	Exon 5 skipping
C0282-10	*r*.70_468del; *r*.1194A>G	Exons 3, 4, and 5 skipping
C0282-11	*r*.70_468del; * r*.1194A>G	Exons 3, 4, and 5 skipping
C0282-12	*r*.213_468del; 1311_1312ins1311+102_1312+176; * r*.1194A>G	Exons 4 and 5 skipping
C0282-13	*r*.213_468del; *r*.993_1018del; *r*.1194A>G	Exons 4 and 5 skipping
C0282-14	*r*.213_468del; *r*.1194A>G	Exons 4 and 5 skipping
C0282-15	*r*.213_468del; *r*.1194A>G	Exons 4 and 5 skipping
C0282-16	*r*.70_468del; *r*.1194A>G	Exons 3, 4, and 5 skipping
C0282-17	*r*.213_468del; *r*.755_848del; *r*.1194A>G	Exons 4, 5, and 8 skipping
C0282-18	*r*.213_468del; *r*.1194A>G	Exons 4 and 5 skipping
C0282-19	*r*.70_468del; *r*.1194A>G	Exons 3, 4, and 5 skipping
C0282-20	*r*.213_468del; 1311_1312ins1311+102_1312+176; *r*.1194A>G	Exons 4 and 5 skipping
C0282-21	*r*.213_468del; *r*.1194A>G	Exons 4 and 5 skipping
C0282-22	*r*.213_468del; *r*.755_848del; *r*.1194A>G	Exons 4, 5, and 8 skipping
C0282-23	*r*.213_468del; *r*.755_848del; *r*.1194A>G	Exons 4, 5, and 8 skipping
C0282-24	*r*.213_468del; *r*.1194A>G	Exons 4 and 5 skipping
C0282-25	*r*.213_468del; *r*.1194A>G	Exons 4 and 5 skipping
C0282-26	*r*.213_468del; 1311_1312ins1311+102_1312+176; * r*.1194A>G	Exons 4 and 5 skipping

The ASVs in this table were described according to the nomenclature guidelines [26, 27]; nucleotide numbering was based on cDNA sequence (GenBank: NM_014251), with +1 indicating the A of the ATG-translation initiation codon.

**Table 3:** *SLC25A13* ASVs harboring *r*.329_468del in the patient C0282 and 8 healthy volunteers.

Subjects	*SLC25A13* ASVs		*χ* ^2^	*P*
	With *r*.329_468 skipping	Without* r*.329_468 skipping		
C0282	26 (100%)	0 (0.0%)	129.2	<0.01
Volunteers	1 (0.9%)	115 (99.1%)		

Correction for continuity was performed for *χ* ^2^ calculation in this table.

## Abbreviations

NICCD: Neonatal intrahepatic cholestasis caused by citrin deficiency
*SLC25A13*: Solute carrier family 25, member 13
DNA: Deoxyribonucleic acid
cDNA: Complementary deoxyribonucleic acid
SNP: Single nucleotide polymorphism
PCR: Polymerase chain reaction
ASVs: Alternative splicing variants
CTLN2: Adult-onset citrullinemia type 2
FTTDCD: Failure to thrive and dyslipidemia caused by citrin deficiency
LA-PCR: Long and accurate-polymerase chain reaction
PCR-RFLP: Polymerase chain reaction-restriction fragment length polymorphism
PBLs: Peripheral blood lymphocytes
MCTs: Medium-chain triglycerides
RT-PCR: Reverse transcriptional polymerase chain reaction
LSM: Lymphocyte separation medium
EDTA: Ethylene diamine tetraacetic acid
RNA: Ribonucleic acid
MMLV: Moloney murine leukemia virus
ORF: Open reading frame
CDS: Coding sequence
AST: Aspartate aminotransferase
GGT: Gamma-glutamyl transpeptidase
Tbil: Total bilirubin
Dbil: Direct bilirubin
Ibil: Indirect bilirubin
TBA: Total bile acids
AFP: Alpha-feto protein
GC-MS: Gas chromatography mass spectrometry
4HPL: 4-Hydroxyphenyl lactate
MS-MS: Tandem mass spectrometry
NMD: Nonsense-mediated mRNA decay
AGC: Aspartate/glutamate carrier
NADH: Reduced form of nicotinamide adenine dinucleotide
NAD^+^: Nicotinamide adenine dinucleotide
UDP-galactose-4-epimerase: Uridine diphosphate-galactose-4-epimerase.
